# Current Trends and Opportunities for Competency Assessment in Pharmacy Education–A Literature Review

**DOI:** 10.3390/pharmacy7020067

**Published:** 2019-06-18

**Authors:** Hayley Croft, Conor Gilligan, Rohan Rasiah, Tracy Levett-Jones, Jennifer Schneider

**Affiliations:** 1 School of Biomedical Sciences and Pharmacy, Faculty of Health and Medicine, The University of Newcastle, Callaghan, NSW 2308, Australia; 2 School of Medicine and Public Health, The University of Newcastle, Callaghan, NSW 2308, Australia; Conor.gilligan@newcastle.edu.au; 3 Western Australian Centre for Rural Health, University of Western Australia, Geraldton, WA 6530, Australia; Rohan.rasiah@uwa.edu.au; 4 Faculty of Health, University of Technology Sydney, Ultimo, NSW 2007, Australia; Tracy.Levett-Jones@uts.edu.au; 5 School of Medicine and Public Health, The University of Newcastle, Callaghan, NSW 2308, Australia; Jennifer.Schneider@newcastle.edu.au

**Keywords:** assessment, health professionals, pharmacy, pharmacy student, pharmacist, competency-based education, simulation, Entrustable Professional Activities (EPAs), Objective Structured Clinical Examination (OSCE)

## Abstract

An increasing emphasis on health professional competency in recent times has been matched by an increased prevalence of competency-based education models. Assessments can generate information on competence, and authentic, practice-based assessment methods are critical. Assessment reform has emerged as an academic response to the demands of the pharmacy profession and the need to equip graduates with the necessary knowledge, skills and attributes to face the challenges of the modern workforce. The objective of this review was to identify and appraise the range of assessment methods used in entry-level pharmacy education and examine current trends in health professional assessment. The initial search located 2854 articles. After screening, 36 sources were included in the review, 13 primary research studies, 12 non-experimental pharmacy research papers, and 11 standards and guidelines from the grey literature. Primary research studies were critically appraised using the Medical Education Research Study Quality Instrument (MERSQI). This review identified three areas in pharmacy practice assessment which provide opportunities for expansion and improvement of assessment approaches: (1) integrated approaches to performance assessment; (2) simulation-based assessment approaches, and; (3) collection of validity evidence to support assessment decisions. Competency-based assessment shows great potential for expanded use in pharmacy, but there is a need for further research and development to ensure its appropriate and effective use.

## 1. Introduction

Assessment is a multi-faceted function that has a powerful influence on learning [1]. Assessment is widespread in health professional education, motivated by accountability to external sources such as accrediting authorities or institutions that wish to improve services and programs [2]. Huba and Freed define assessment as “the process of gathering and discussing information from multiple and diverse sources in order to develop a deep understanding of what students know, understand, and can do with their knowledge as a result of their educational experiences [3]. This process is realised when assessment results are used to improve subsequent learning” [3]. This definition of assessment is particularly useful as it highlights important characteristics of quality assessment processes: (1) a systematic and continuous process; (2) having emphasis on student learning, focusing on what students can *do*, and (3) focusing on improvement of future performance (for individual students), and of educational programs [2,3]. There are several reasons documented to justify the increasing emphasis on the assessment of health professionals, as outlined in Table 1.

Traditionally, assessments have been used to make summative evaluation decisions, serving as a concrete and clear approach for educators to assure external stakeholders of the competence of students, often referred to as Assessment of Learning (AOL). While feedback has always been part of the assessment process, in the last decade, educators have recognised the positive impact that formative assessments can have on student learning [11]. Indeed, there is now broad agreement among professional associations, accrediting agencies and educational institutions that student learning should be a primary goal of assessment [2,11]. This is evidenced by the increasing emphasis on assessment for learning (AFL), an approach using formative assessment or informal assessment approaches to specifically improve students’ learning [11,12]. Current literature insists that assessment should not be dichotomised into approaches that are either formative or summative [11]. Examples of assessment frameworks that combine both purposes, include the Objective Structured Clinical Examinations (OSCE) which may include an overall global assessment, as well as assessing different skill sets in a deconstructed manner that can be leveraged to provide feedback to students on their strengths and weaknesses [13,14].

A large body of evidence demonstrates the need for multiple assessments throughout a student’s learning trajectory, rather than a single high-stakes capstone assessment, to ensure students build towards minimum acceptable level of knowledge or performance [12,15,16]. This has been reinforced through the evolution of competency-based medical education literature [17]. While there is little argument about the importance of using competencies to frame educational outcomes, there is less certainty around how we equate competency statements and frameworks into relevant measures of professional practice [18]. Similarly, approaches for the design and development of trackable paths for students to ultimately reach independent and/or advanced practice are not widely established. Entrustable Professional Activities (EPAs) offer promise in this area and are currently a focus of educational research in pharmacy [19,20]. Furthermore, capstone courses in degree programs with multiple comprehensive and integrated student assessments are emerging and have been shown to provide faculty members with feedback regarding curriculum outcomes attained through robust assessments [21].

In an attempt to maintain and improve the quality and safety of patient care, a framework for assessment of work-ready pharmacy graduates should promote the application of expertise and professional judgement of technical and non-technical skills in areas of practice that have the potential to impact patient safety, such as medicine dispensing [22]. However, despite the widespread interest in competency-based education programs and practice-based assessments, there is a scarcity of literature reporting on valid and reliable assessment instruments in pharmacy education. Here, we attempt to provide an overview of assessments used to evaluate the competence of pharmacists graduating from their degree program and entering the pharmacy profession.

## 2. Background

The medical education field has been utilising competency-based assessment approaches for several years [23], and these approaches are now beginning to be adapted and implemented in pharmacy education [24]. The competency-based approach is learner-centred and is underpinned by enabling the progression of students who demonstrate adequate knowledge and/or skills in assessments developed within the program of study, while simultaneously preventing students from graduating without demonstrating the required level of competence [24]. A formal competency-based education (CBE) model removes traditional semester timeframes as the yardstick for determining readiness and enables students to learn and progress at their own pace [24]. However, given various practicalities, attention is currently still focused on students demonstrating competencies during the traditional time-limited and structured curricula. Both approaches highlight the increasing need for adequate assessment, and why the concept of EPAs is growing in popularity as an approach to ensure students possess the required skills, knowledge and attitudes both prior to, and beyond program completion.

Outcome-based education in pharmacy programs is evolving to embrace the competency-based assessment frameworks set forth by national and international governing bodies. These frameworks are increasingly used to describe the skills pharmacists require to effectively meet the health needs of patients. The International Pharmaceutical Federation (FIP) states that assessment and quality assurance is the key to guarantee student capabilities, a recommendation that would be difficult to achieve without a competency-based approach [25]. For example, The Center for the Advancement of Pharmacy Education (CAPE) provide educational outcomes that focus on the knowledge, skills, and attitudes that entry-level pharmacists require. The CAPE learning objectives provide a structured framework for measuring the outcomes of a degree program in pharmacy, including those that are necessary for the safe and appropriate supply of medicines [26,27]. Some other examples include National Competency Standards Framework for Pharmacists in Australia [28] and Standards for the initial education and training of pharmacists in Great Britain [29]. In an international review of the use of competency standards in undergraduate pharmacy education, it was shown that competency standards were reflected in pharmacy program assessments, particularly OSCEs and portfolios, as well as being used to design, develop and review pharmacy curricula [9]. While this provides a quality assurance mechanism to enhance the quality and employability of the final graduate, we must be cognisant of the limitations of translating these competency frameworks into the tasks that are activities of daily Pharmacy practice when designing assessments [18,30].

While competency-based education is evident in the field, the challenges associated with competence assessment have not been fully resolved. Broader cooperation in research and practice is required to ensure validity and authenticity of competence-based education and assessment internationally [2].

## 3. Review Methodology

### 3.1. Aim

The following review aims to identify opportunities for future research in assessment of entry-level pharmacists.

### 3.2. Methods

Medline, International Pharmaceutical Abstracts (IPA), EMBASE, CINAHL, PsycINFO and Scopus were searched for articles in English published between January 2000 and May 2019, to identify studies that reported on the use of assessments in pharmacy education. The literature search was performed iteratively, with broad search terms used initially, primarily focusing on teaching and assessment in health professional education. The following key search terms were used: clinical assessment [assessment OR evaluation OR measurement OR competence OR standard, OR outcomes OR entrustment OR workplace OR preceptorship OR placement OR work-integrated learning], assess*, studen*, educ*, health professional student [students OR undergraduates]. Subsequently, additional terms and filters were added to increase the sensitivity and specificity of our search to pharmacy education. These include pharm*, “assessment, pharmacy”, “education, pharmacy”, “practice, pharmacy”.

Thirteen experimental research studies specific to pharmacy assessment approaches were identified, and these are outlined in Table 2. Studies were predominantly data-based evaluations of assessment practice, including validity studies, with some comparisons of assessment practice. Quantitative research papers were appraised using the Medical Education Research Study Quality Instrument (MERSQI). This instrument assesses studies’ according to (1) Design; (2) Sampling; (3) Type of data; (4) Validity evidence for evaluation instrument; (5) Data analysis; and (6) Outcome.

**Figure 1:**
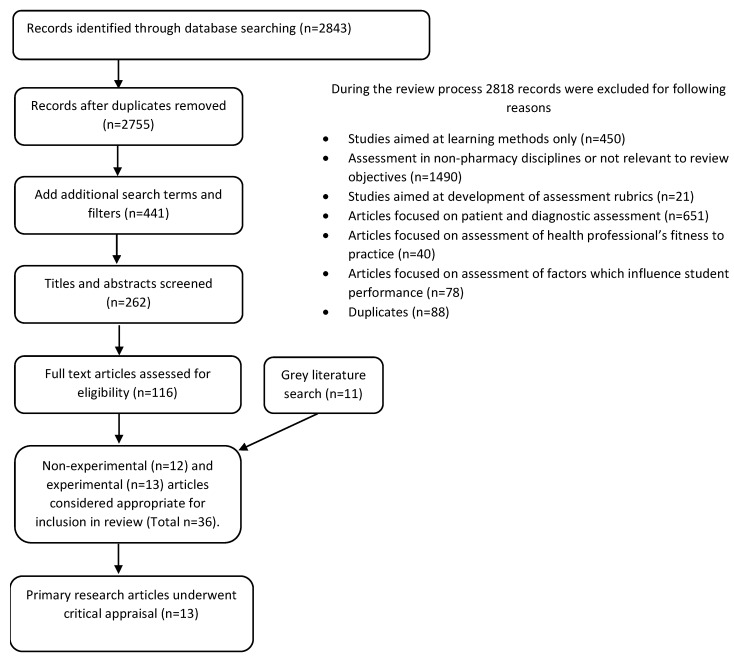
PRISMA Flow Diagram for the literature review search [32].

Further, 12 non-experimental pharmacy research papers (commentary, review, editorial, practice applications) were identified and drawn upon in the following discussion. A grey literature search was completed, and 11 relevant documents were identified including competency frameworks, standards and policies that inform assessment practice. This gave a total of 36 relevant literature sources pertaining to assessment in pharmacy. Millers Pyramid has been used as a tool against which we compare the application of various assessment methods [15,31]. The Preferred Reporting Items for Systematic Reviews and Meta- Analysesis (PRISMA) approach has been used for reporting on the literature search as shown in Figure 1. For completeness we have drawn on research completed in other health disciplines to develop a wider appreciation of the trends in health professional education. Therefore, in addition to pharmacy literature, 49 relevant papers from other health professional fields were used to inform the analysis and interpretation of our findings.

### 3.3. Inclusion/Exclusion Criteria

Articles were included if they reported in English language on assessment approaches used in pharmacy education. Reasons for exclusion are shown in Figure 1.

### 3.4. Method of Analysis

A data reduction process was used to extract, simplify and organise data from the articles. Each article was analysed to identify data relevant to the objective of this review, and this information was recorded. The data categories were refined iteratively as the literature was analysed. Thematic analysis was then used to capture ideas and concepts that recurred across the data set to develop themes.

## 4. Results

The review results presented describe the variety of assessments used in pharmacy education and mapped to the levels in Miller’s Pyramid [15] (Table 2 and Table 3); as well as describe the included experimental research studies (n = 13) pertaining to assessment in pharmacy education (Table 4). Across 13 studies, the mean overall MERSQI score was 10.6 (range 5–15, of possible 18).

The implications for current and future pharmacy education practice are then synthesised in a discussion of key themes emerging from the results and drawing on the wider literature for health professional assessment. Three main themes relating to competence assessment in pharmacy education were identified in this literature review: (1) integrated approaches to performance assessment; (2) authentic and simulation-based assessment approaches, and; (3) collection of validity evidence to support assessment decision.

The articles retrieved from this review collectively describe the pedagogical challenges faced by educators in their efforts to evaluate pharmacist competence in their activities of daily practice, such as medicine dispensing which represents a core activity of daily pharmacy practice [63]. Dispensing is the process of preparing and supplying a medicine for use by a patient, in a manner that optimises its effectiveness and safety [64,65]. Dispensing practice by pharmacists has traditionally taken a product-orientated approach but has more recently been extended to incorporate a more patient focused clinical role incorporating communications with patients and other health professionals, medicines reconcilliation and review of clinical data [63,65,66]. Indeed, pharmacy practice has evolved to incorporate a variety of professional tasks in designing, implementing and monitoring a therapeutic plan to optimise therapeutic outcomes for the patient, and it is imperative that competence assessment evolves to meet these demands [66].

This review highlights six key challenges in the assessment of health professionals that impact evaluation of pharmacists’ professional skills, grouped under the themes identified from the literature review.

**Theme** **1.** *Authentic, simulation-based assessment approaches.*

### 4.1. Achieving Authenticity in Assessment Tasks and Activities

A shift from the application of a predominantly behavioural pedagogy to a constructivist learning paradigm has placed a much greater emphasis on authentic teaching and learning practices [67]. This has led to authentic assessment strategies becoming a focal strategy in higher education over the last two decades, aimed to provide a connection between performance and real-world work environments. However there is not always consensus on what constitutes authenticity in assessment [54].

In the past two decades, there has been a significant increase in the use of simulation-based training and assessment as part of the education of competent and safe healthcare professionals [68,69], including pharmacists [70,71,72,73]. Simulation refers to an artificial representation of a real-world process to achieve educational goals through experiential learning [74]. Simulation-based training and assessment activities are those that use simulation methodology to recreate a clinical scenario that replicates key aspects of an actual patient encounter, and provide opportunities for students to learn and practice skills in a safe environment with no risk of harm [75]. The increased use of simulation for teaching and assessment in healthcare education has been driven by a range of factors, including increased student numbers and changes in the structure of both academic programs and healthcare delivery. This has been compounded by a decrease in the availability of patients for educational opportunities, and the need to standardise the clinical situation faced by students for assessment purposes [68,69,76]. Simulation applications in assessments range from high fidelity, technical simulations with manikins to role-plays with simulated patients [72,73], however reports on such assessment approaches in pharmacy education to evaluate learner readiness for patient care in the clinical environment are limited.

**Theme** **2.** *Collection of validity evidence.*

### 4.2. Reporting on the Validity and Reliability of Assessment Methods

Educators and accrediting bodies for health professionals are required to use various types of assessment and examine the validity and reliability of evaluation methods used [77,78,79]. Validity and reliability of assessment methods is pivotal in being able to consistently produce valid judgements based on assessment information [37]. For this reason, the selection and construction of appropriate metrics to evaluate competence, minimisation of measurement errors and validation procedures are currently major focus areas in health professional education [69,76]. What is less clear from our review, is what is required in measurement models to enable assessors to move from using inferences obtained from simply observing the actions and behaviours of students, to having clear evidence for a student’s skills, strategies and proficiencies, based on a valid and reliable framework, and this represents opportunity for future research.

The process of validation has evolved significantly, and our understanding of validity theory, as it relates to assessment in health professional education, has become increasingly complex. This is further complicated by ongoing transformations of health education curricula which support a move towards competency-based, programmatic assessment and authentic assessment methodologies [80]. As we move towards an increased reliance on performance-based assessments for decisions about health professionals’ readiness and ability to perform in the workplace, there is a need to defend the processes used and decisions made, particularly for high-stakes assessment.

### 4.3. Selection of Appropriate Assessment Metrics

Analytic and holistic scoring are two common approaches to assessment, although these assessment scales are not always mutually exclusive, in that analytic scoring may also influence an overall holistic rating. Checklists are one of the most common methods used for analytic scores [81,82,83,84,85,86,87], constructed with items that measure specific steps or processes across varying domains that include history taking, patient counselling and use of clinical devices (e.g., questions that should be asked, manoeuvres that should be performed). Although checklists provide modestly reproducible scores and have good internal validity, there are some drawbacks to this methodology [76,82]. A checklist-based assessment could alter the behaviour of the candidate, who may employ rote-learned behaviours or alter the sequence or timing for a task, in order to accrue more points. A checklist may also ignore important factors relating to the assessment such as the order and timing of actions, which may be critical where a series of patient management activities needs to be employed [76].

Conversely, holistic scoring can effectively measure complex and multidimensional constructs such as communication and teamwork. The psychometric properties of holistic scoring are often adequate [76], and one of the key benefits is that they allow the rater to evaluate implicit actions of the candidate, which are unnecessary or have a negative impact on patient care, something that is difficult to achieve with the use of a checklist. Global rating scores are the prevailing methodology for holistic scoring, whereby a multitude of performance factors are considered in an overall rating or evaluation of health professional competence [86,88,89,90,91,92]. Challenges for the application of global rating scales include inter-rater standardisation and appropriate consideration of the individual elements contributing to the global scale. Within pharmacy education there is an absence of integrated assessment models that enable a student to be assessed holistically, where the entire performance can be rated.

### 4.4. Accounting for Different Levels of Ability and Practice

Another of the challenges in competence assessment is avoiding a ‘one-size-fits-all’ approach [93]. Often, assessment tools are standardised using competency standard frameworks, and fail to take account of differing levels of knowledge, skills and experience in health practitioner development. For example, the same competency assessments may be used in an undergraduate pharmacy program, postgraduate training, newly employed pharmacists, ongoing CPD and advancing practice without adequate alteration of the level of expectation or difficulty of the task. This ‘tick-box’ approach where the same competency tool is used among all pharmacists is pedagogically unsound [93]. This raises questions about the validity and reliability of generic assessment tools that are expected to fulfil the assessment requirements of all health professionals in different clinical contexts.

Although focusing primarily on knowledge assessment, computer-adaptive tests (CAT) have been increasingly used to customise the assessment process using advances in technology [37]. CATs are designed to adjust their level of difficulty based on the responses provided, to match the knowledge and ability of the test candidate and are intended to be appropriately challenging for each student [94,95]. This approach may overcome issues associated with ‘one-size-fits-all’ standardised tests. For example, a candidate that answers a question correctly will then be given a question that is more difficult, with scoring that not only considers the percentage of correct responses, but also the difficulty level of items completed [37,95].

**Theme** **3.** *Integrated approaches to assessment.*

### 4.5. Integration of Competencies in Assessments

Medicine dispensing by pharmacists is commonly assumed to be a simple, routine process. However the dispensing process is underpinned by multiple, parallel steps that include interpreting and evaluating a prescription, retrieval and review of a medication history, selection, preparation, packaging, labelling, record-keeping, and transfer of the prescribed medicine to the patient, including counselling as appropriate [63,96]. The dispensing process may also incorporate other associated tasks such as communication with the prescriber, and provision of more complex advice to the patient [96]. These steps are often assessed in isolation [55,56,58], however in the practice environment, these tasks are frequently performed concurrently, whereby a pharmacist performs two or more of these actions simultaneously, to complete the process as a single encounter, with elements rarely performed in isolation [97].

OSCE/simulation hybrid assessments are those which incorporate simulation methodology into one or more OSCE stations, where each station is built around a specific task or specific component of the more extensive process. This generates information about several dimensions of competence, which may be integrated by combining stations where one builds on another. This blended-simulation methodology has been used successfully in Pharmacy education, most recently in a study evaluating patient care outcomes in first- and third-year pharmacy students and postgraduate pharmacy residents as an overall assessment of practice readiness [63]. It is unclear, however, how well such blended assessment approaches represent a students’ competency to perform the task as an integrated whole.

The OSCE is commonly used as an acceptable way of evaluating clinical skills in undergraduate medicine and pharmacy programs worldwide, as it can facilitate assessment across a wide range of clinical contexts [37,98]. This method of examination enables the rater to tease out deficiencies in specific competency areas, thus providing feedback for the students’ progress, particularly during the early stages of learning. The emergence of ‘reductionism’ in assessment has led to the breakdown of the competencies we wish to evaluate into smaller, discrete tasks, which are then assessed separately. However, mastery of the parts does not automatically imply a competent performance of the integrated whole process being examined. The traditional OSCE format tends to compartmentalise candidates’ skills and knowledge and fails to assess some important multidimensional domains of pharmacy practice including integration of core knowledge into practice, clinical reasoning, multi-tasking and time-management.

EPAs by definition are statements of specific task-related activities that require integration of multiple competencies [18,37], e.g., ‘gather a history and assess a patient’s current medication regimen to ensure medications are indicated, effective, safe, and convenient’ [18] and therefore play an important role in ensuring we view competence as an integration of knowledge, skills and attitudes into a practice-orientated situation. The most extensive example of the application of the EPA model to pharmacy education is in the Doctor of Pharmacy curriculum, USA, an entry-level pharmacy degree. The American Association of Colleges of Pharmacy (AACP) has published 15 core EPAs essential for all pharmacists to perform without supervision as entrusted by stakeholder groups [99,100], and these EPAs have been used to develop an assessment framework across the Advanced Pharmacy Practice Experiences (APPE) curriculum in the United States [18]. The APPE comprise the fourth and final year of the Doctor of Pharmacy curriculum, commonly referred to as ‘rotations’ where students participate solely in experiential learning. While on APPE rotations students can be assessed using the 15 core EPAs. There is current effort to include the same approach within the Introductory Pharmacy Practice Experiences (IPPEs) across the earlier years of the curriculum. Beyond this, despite the increasing momentum for EPAs as a novel framework for competency-based pharmacy education, including the attainment of educational outcomes [18,26], other examples are more isolated small scale trials, or only limited to use in specific parts of a pharmacy program such as capstone course components [101,102]. No evidence supporting the effectiveness of an EPA framework in Pharmacy education has been published. Additionally, EPAs have not yet been used widely in pharmacy undergraduate education and further research and collaboration is needed to implement this approach more extensively [20].

### 4.6. Assessment of Cognitive Processes

Assessment of medicine dispensing skills can be challenging because it involves complex clinical and inter-relational judgements, rather than simply a series of technical or psychomotor actions to determine clinical competence. Clinical judgements relating to the safety and appropriateness of medicine are complex and dynamic, underpinned by several cognitive processes including retrieving and reviewing information, processing information, identifying issues, collaborative planning, decision-making and reflection [63,103]. Therefore, practitioner competence can be difficult to measure. Similar pedagogical challenges are documented in other professions [93,104]. A holistic approach to competence assessment is gaining momentum due to its ability to blend a range of processes that underpin clinical reasoning, however further work to incorporate this approach into assessment is needed [2,105,106].

## 5. Discussion

This review investigated the utilisation of competency assessment tools in pharmacy education. While there is no gold-standard assessment methodology, three key themes have been identified which provide opportunities for future development and research: (1) integrated approaches to performance assessment; (2) authentic and simulation-based assessment approaches, and; (3) collection of validity evidence to support assessment decisions.

The competency movement in medical education argues that an integrated approach to competence [16], and an integrated skills assessment, which evaluates a candidate’s ability to view the patient and dispensing process holistically, is more suitable during the advanced stages of training [37,44,107,108,109]. EPAs provide a mode for integrated approaches to assessment in pharmacy education, and although there is a strong theoretical basis for using EPAs to assess the performance during workplace-based training, less is known about how entrustment decisions can be incorporated into other assessment types and settings.

Our understanding of the utility of simulation-based assessments to offer an authentic and integrated skills assessment is increasing [77]. The most recent systematic review of simulation-based assessments in healthcare focused on ‘technical’ skills only which is indicative of a gap in the literature in relation to the use of simulation for the assessment of ‘non-technical’ skills, including interpersonal communication, teamwork and clinical decision-making [69]. There is a general consensus on the need to do further research into simulation-based assessment methods that focus on non-technical skills, particularly in studies in nursing [90,91,110,111,112]. Most simulation in pharmacy uses blended simulation in an OSCE context. Outside of the traditional OSCE format, there are a lack of studies specifically pertaining to the integrated skills assessment of pharmacists or pharmacy students’ competence, and fully integrated simulation assessments have not been reported. An opportunity exists to develop and evaluate integrated skills assessment that requires the candidate to manage all aspects of the patient encounter, incorporating important skills for clinical work including multitasking and task switching [97].

Although there are examples of validated tools in pharmacy education [47], many have been validated based on psychometric properties alone. There is increasing understanding that the isolated use of such approaches to the validation of current trends in complex assessment models may give an incomplete evaluation of the quality of the assessment overall [80,113,114]. More work is needed to ensure evidence-based validation approaches with multiple sources of evidence are used to support the proposed score interpretations, including the type of evidence collected, depending on the assessment instrument in question, and its intended application.

## 6. Conclusions

A dichotomy in the purpose of assessment is clear: accountability and student learning. While the concept of competency-based education is growing internationally, the future is not certain, and more work is required to ensure a valid and reliable measure of competency. This review of literature pertaining to assessments in pharmacy education is particularly useful in identifying opportunities to work towards the development of valid and reliable assessment frameworks. The goal of such frameworks is judgements which represent a valid reflection of the level of competence of pharmacy students and pharmacists. The review shows a need exists to explore key areas of assessment in pharmacy education, including: (1) integrated approaches to performance assessment; (2) authentic and simulation-based assessment approaches, and; (3) collection of validity evidence to support assessment decisions. There is a scarcity of published literature demonstrating the use of truly integrated simulation-based assessments in pharmacy. The results of this review provide motivation for further developing integrated assessment methods for assessing the competency of future pharmacists.

## Figures and Tables

**Table 1:** Reasons for change in focus on practice-based assessments in health professional education.

A move towards outcome-based education models, including competency-based approaches [2,4]
Increased quality assurance (QA) of tertiary education, evidenced directly through student performance [5]
Emphasis on human factors implicated in medical error and patient safety [6,7]
Increasing government and community expectations and pressure on universities for ‘work-ready’ graduates [8] including increasing attention for ‘registration upon graduation’ (RUG) models such as in the US and Thailand compared with ‘degree plus professional registration’ models common to the UK, Australia and New Zealand [9]
Increased accreditation requirements for programmes [10]
Integration of professional competency standards into education programs [2,9]
Increasing employer expectations [8,9]

**Table 2:** Description of assessments used in pharmacy education.

Assessment Process	Description	Assessment Characteristics Related to Literature Review Themes		
		Integration of Competencies	Authenticity	Validity and Reliability
Multiple-Choice Questions (MCQs) including Extended Matching Questions (EMQs) and computer-adaptive tests (CATs)	Traditional MCQ most widely consists of a question (stem) followed by several (typically 4–5) possible answer options; may also be true/false format [33]. Single-best option MCQ format has been used extensively as a method of assessment [33]. EMQs are organised into four parts; a theme; a list of possible answers (options); question (lead-in statement); and a clinical problem (Stem) [34]. CATs select items for candidate based on their previous response and therefore customise assessment process according to ability.	Primarily assess knowledge in specific subject areas; may be possible to assess higher cognitive processes (e.g., interpretation, knowledge application) with well-constructed clinical scenarios [35]. EMQs are superior to traditional MCQ in assessing problem solving and clinical reasoning abilities [34].	Lack assessment authenticity [33]; encourage rote learning [33].	High levels of reliability [33,35]. Validity will vary depending on content and construction of questions, number of answer items. Validity may be improved by implementing training, writing guidelines, peer review and other validation processes [36]. Evidence suggests three-option MCQs improve both assessment efficiency and content validity.
Written examination, including modified essay question (MEQ)	Traditional written examination usually requires candidates to respond to a variety of questions using short, long or (mini/modified) essay style; open-ended responses in written form. Questions may elicit specific knowledge or facts, or incorporate theory of clinical skills and communications.	Primarily assess knowledge in specific subject areas; may be possible to assess higher cognitive processes (e.g., interpretation, knowledge application) with well-constructed clinical scenarios [35]. ‘Open-book’ written examinations also assess ability to incorporate assessment of information retrieval and incorporation into response.	Generally, lack authenticity, as students are not able to demonstrate performance. Questions that assess application of knowledge in real-world scenarios are more authentic than those that focus on student’s ability to reproduce information.	Lower levels of reliability when compared with MCQ, since responses are open ended. Wider sampling and more directed questioning generally increase reliability. Validity will vary depending on content and construction of questions, number of answer items.
Viva Voce “viva”/traditional oral examination	Oral (rather than written) examination conducted face-to-face with examiner(s).	As well as clinical knowledge, viva may be useful for the assessment of characteristics which are difficult to assess via other techniques such as professionalism, clinical reasoning, ethics, communication skill, problem solving [37].	Lack assessment authenticity due to the hypothetical nature of questioning.	Viva examinations are often unstructured, wide variation may occur between questioning for different candidates and different assessors, thus they are prone to errors of variability [35]. Inter-rater reliability is generally poor [38] and validity is difficult to establish, dependent on the content of the questions asked, however given the flexibility in being able to vary content has the potential to improve validity [38].
Simulated patient encounters/“role plays” and practical examination	Examination of practice-based skills through demonstration of that task e.g., patient counselling, pharmaceutical compounding examination [21].	Tend to focus on one area of practice e.g., preparation of compounded product, counselling, medication history taking [39].	Has the potential to be authentic. Authenticity is increased when psychological fidelity is high and decision-making closely simulates the real context of the skill [40] and requires integration of competencies.	Examples of validity and reliability established for some tools including communication and counselling skills of pharmacists (CCSP) tool [39] and medication related consultation framework (MRCF) [41]. Validity evidence presented in the literature relies heavily on psychometric properties.
Objective Structured Clinical Examination (OSCE)	The OSCE objectively tests multiple skill sets in a controlled environment. Candidates move through a series of time-limited stations for the assessment of professional tasks in a simulated environment using standardised marking rubrics [37,42].	Encourage students to practice skills more holistically [37]. However, OSCEs tend to break down competencies into smaller units which are evaluate separately. Lack complete integration of clinical tasks.	Although OSCEs often use trained simulated patients in simulated environment, authenticity has been questioned as scenarios may not reflect the reality of clinical practice [43].	Validity and reliability should be established for individual assessments. However, increasing the number of stations can improve validity and reliability [42], while increasing time spent at each station, using a standardised marking tool, use of standardised patients and having multiple assessors at each station can improve reliability [42].
Workplace Based Assessments (WPBAs)	Case-based discussion (CBD) [44], Direct observation including mini-Clinical Evaluation Exercise (CEX) and multisource/360-degree feedback [45,46] are all assessment tools that evaluate performance in the environment in which the practitioner works [37].	Integration is dependent on the assessment.	Authenticity is high due to the assessment of competence and performance takes place during normal work activities; advantages of authenticity rely on the appropriate use of tools, and engagement of both learner and assessor [37].	Validity and reliability should be established for individual assessment tools. Often content validity is limited, as the assessment is of the student’s management of one specific case at one point in time. Construct validity is high because the tools assess actual practice in the workplace. Reliability is often dependent on assessor’s training and experience, and may be improved by using standardised, validated assessment tools. Examples of validated assessment tools in pharmacy education, e.g., pharmacy mini-PAT [47]; and in other health disciplines e.g., SPRAT [48].
Portfolio	A collection of longitudinal evidence of professional development including performance evaluation samples, action plans, self-reflection, evidence of continuing professional development (CPD), presentations, documentation of critical incidents, evidence of research and quality improvement projects.	Integration is dependent on the source of evidence in the portfolio; there is opportunity to capture evidence from a range of settings that show amalgamation of competencies, but content requirements of portfolio may need to be clearly defined to ensure this.	Authenticity is high as samples of evidence are directly from workplace; may be used as a repository for completes WPBAs.	Valid method for assessing competence, however threats to validity exist as contents may vary considerably and are self-reported. Evidence shows a wide range of reliability scores [49].
Entrustable Professional Activities (EPA)	EPAs are used as both a link competencies and professional responsibilities in practice; and as a mechanism to decide the level of supervision for a student [20].	High level of integration as EPAs require multiple competencies to be applied in an integrative fashion [50], e.g., clinical tasks such as medicine dispensing combines several domains of competence [51].	Authenticity is high due to the assessment of competence and performance takes place while performing units of professional practice that reflect the daily work of the practitioner.	Few studies report on the psychometric properties of EPAs. Those that do report moderately strong inter-rater reliability [52] and good face validity.

**Table 3:** Mapping of assessment approaches to Millers Pyramid.

Assessment Type	Millers Pyramid Level [15]				
	Knows (Knowledge)	Knows How (competence)	Shows How (Performance)	Does (Action)	Is (Identity)
Multiple-Choice Questions (MCQ)	Yes	Partially	No	No	No
Extended Matching Questions (EMQ)	Yes	Partially	No	No	No
Written Examination	Yes	Yes	No	No	No
Computer-Adaptive Testing (CAT)	Yes	Partially	No	No	No
Viva Voce/Oral Exams	Yes	Yes	Partially	No	No
Simulated patient encounters/practical examination	Yes	Yes	Partially	No	No
Objective Structured Clinical Examination (OSCE)	Yes	Yes	Yes	No	No
Workplace Based Assessments (WBA)	Yes	Yes	Yes	Yes	Yes
Portfolio	Yes	Yes	Yes	Yes	Yes
Entrustable Professional Activities (EPA)	Yes	Yes	Yes	Yes	Yes

**Table 4:** Description of the included experimental research studies in pharmacy assessment (n = 13) and quality appraisal using the Medical Education Research Study Quality Instrument (MERSQI) [53].

Citation/Location/Quality	Study Participants/Assessment Approach	Study Aims/Methods	Outcomes and Key Findings	Limitations	Reports on Integration of Competencies (Y/N)	Includes Simulation/Reports on Authenticity (Y/N)	Measures Validity/Reliability of Assessment Tool (Y/N)
Santos, S and Manuel, J. (2017) [54] Brisbane, Australia 5/18 (MERSQI)	Fourth-year undergraduate BPharm students (n = 14) and assessors (n = 6). Demonstration of research skills via submission of an abstract, poster and oral presentation.	Aim: Describe and evaluate the design and implementation of an authentic assessment in undergraduate pharmacy course. Survey of students and stakeholders’ perceptions on authenticity of assessment.	Authenticity in assessment is subjective to each student. Authenticity as rated by students was perceived as lower when compared with assessor perceptions. Use of a framework in design of an authentic assessment is valuable.	Single site. Small sample size. Level of details in statistical reporting poor.	N	Y	N
Hirsch, A; Parihar, H. (2014) [21] Georgia, USA 12/18 (MERSQI)	Fourth-year undergraduate PharmD students (n = 73). Broad range of assessment tools incorporated into a mega-OSCE (to evaluate student knowledge and skills written and verbal presentations, multiple-choice examinations, short answer calculations, standardized patient encounter and pharmaceutical compounding).	Aim: To create a capstone course that provides a comprehensive and integrated review of the pharmacy curriculum. Evaluation of student outcomes based on several assessment tools (components of a mega-OSCE).	95% of students successfully passed the capstone course. Qualitative data described students rated the capstone course highly. Robust assessment techniques allowed faculty members to detect specific weaknesses and enabled remediation of those skills.	Details about individual assessments are poorly described. No control group.	N	Y	N
Mackellar, A. et al. (2007) [55] Manchester, UK 11/18 (MERSQI)	Pharmacy academics across three universities (n = 38). Tool for patients to assess the communication skills of pharmacy students.	Aim: To identify valid and reliable criteria by which patients can assess the communication skills of pharmacy students. Literature review and focus group discussion generated the potential assessment criteria. Survey was subsequently conducted to measure face validity and reliability for each assessment criterion.	7 criteria identified that were important measures of pharmacy students’ communication skills and rated as face valid and reliable. The use of a 5-point descriptor scale (excellent, very good, good, fair and poor) is more discriminating than a 6-point numerical scale.	Limited statistical power due to modest sample size.	N	Y	Y
Kadi, A. et al. (2005) [56] Saudi Arabia 10.5/18 (MERSQI)	First-year undergraduate pharmacy students (n = 38). Assay analysis of compounded product for drug content and compared with nominal concentration.	Aim: To evaluate the accuracy of pharmacy students’ compounding skills. Objective assay result reported as a percentage difference from the nominal concentration.	Errors ranged from 25% to >200% of the label amount. 15% of students required >3 attempts before successfully preparing solution. Use of analytical methods that can be quick and inexpensive are important as an objective measurement of students compounding ability.	Only 54% of students participated. Lack of control group.	N	Y	N
Salinitri, F. et al. (2012) [13] Detroit, USA 10.5/18 (MERSQI)	Third-year undergraduate pharmacy students (n = 54). Objective Structured. Clinical Examination (OSCE) compared with written multiple-choice examination.	Aim: Compare pharmacy students’ performance on an OSCE to their performance on a written examination for the assessment of problem-based learning. Effectiveness of OSCE evaluated by 1) comparing OSCE results with written examination skills; and 2) survey views on effectiveness of OSCEs as perceived by faculty and students.	OSCE performance did not correlate with written examination scores. OSCE’s evaluate different competencies (clinical skills, problem solving, communications, social skills, knowledge) not measured with written examinations (knowledge, problem solving). Process of using OSCE was valued by students and faculty observers.	Single site. Survey tools were not validated. Order effect (OSCE first versus written examination first) not measured and may influence results.	Y	Y	N
Sturpe, D. (2009) [14] Maryland, USA 7/18 (MERSQI)	PharmD faculty members (n = 88). Objective Structured. Clinical Examination (OSCE).	Aim: Describe the current OSCE practices (awareness of, interest in, current practice and barriers) in Doctor of Pharmacy (PharmD) programs in the Unites States. Structured interviews with PharmD faculty members.	37% of program responses reported using OSCEs; 63% of program responses reported they did not use OSCEs, but half of these were considering incorporating it into their curriculum.	Descriptive statistics were used to analyse interview transcripts.	N	N	N
Rastegarpanah, M. et al. (2019) [39] Tehran, Iran 11.5/18 (MERSQI)	Third- and fourth-year undergraduate pharmacy students (n = 12) and faculty experts (n = 7). Standardised patient (SP) simulation encounter.	Aim: Design and validate a tool to assess pharmacy students’ performance in developing effective communication and consultation skills. A 22-item tool was developed, and psychometric properties described following its use in student simulation encounters.	High inter-rater reliability between expert raters and simulated patient (SP) ratings (*p* = 0.01).	Small sample size limits generalisability; no control group. No between-scenario analysis to determine effect of different clinical scenarios.	N	Y	Y
Kirton, SB and Kravitz, L. (2011) [57] Hertfordshire, UK 13/18 (MERSQI)	Recent graduates of undergraduate pharmacy program now completing “preregistration” year (n = 39). Objective Structured. Clinical Examination (OSCE) compared with traditional written examination consisting of a series of multiple-choice and essay questions (MPP3 examination).	Aim: investigate correlation between performance in OSCE and traditional pharmacy practice examinations at the same level. Analysis of grades attained by student in their Year 1, 3 and 4 OSCE with assessment data from a same level Medicines and Pharmacy written examination.	When comparing Year 3 OSCE and Year 3 written exam data there was moderate correlation between results from the two methods of assessment. OSCEs add value to traditional methods of assessment because the different evaluation methods measure different competencies.	Data from OSCEs in Year 2 of the program was incomplete and therefore omitted in the analysis.	N	N	N
Kimberlin, C. (2006) [58] Florida, USA 10.5/18 (MERSQI)	Faculty members primarily responsible for communication skills instruction (n = 47). Standardised patient (SP)/simulation encounter (including video recorded consultations); include self-assessment, peer assessment and faculty/expert assessment.	Aim: Describe current practices in assessment of patient communication skills in US colleges of pharmacy Gathering syllabi and assessment instruments from programs, conducting content analyses of assessment instruments and conducting telephone interviews with academics about assessment procedures used in their institutions.	Content analyses revealed there is considerable variety in the skills assessed and the formatting and weighting of different skills. Qualitative interview data indicated concerns with lack of explicit criteria for acceptable performance and perceived lack of reliability for grading.	Modest (56%) response rate. Details about assessment methodology poorly described.	N	Y	N
Aojula, H. et al. (2006) [59] Manchester, UK 9/18 (MERSQI)	First-year Master of pharmacy (MPharm) students. Online computer-based assessment (CBA) using an online learning environment WebCT (course tools), consisting of multiple-choice questions (MCQ) and longer questions including text-match, diagram labelling and calculations.	Aim: Explore computer-based approaches for summative assessments with emphasis on development time, academic rigor, security and organisation. Pilot study was conducted and scores from CBA were compared with traditional marking of exams.	Discrepancy between hand-marking and computer-based marking was <1%, initially improved by embedding a Spellcheck tool. Online summative assessments may be used successfully with (1) an appropriate learning management system with inbuilt assessment tools; (2) time to familiarize staff to write CBA questions; (3) Training students with formative tests; (4) Contingency plan in case of internet failure.	Single site. No control group. Pilot study with module focused on cell biology and biochemistry not representative of practice-based pharmacy knowledge and skills.	N	N	N
Kelley, K. et al. (2008) [60] Ohio, USA 12/18 (MERSQI)	Fourth-year undergraduate PharmD students (n = 109). Case-based interactive assessment.	Aim: To develop an assessment tool that would (1) help students review therapeutic decision-making and improve confidence in their skills; (2) provide pharmacy practice residents with opportunity to lead small group discussions (3) provide program-level assessment data. Survey to measure student confidence in their skills and knowledge, and perceived usefulness of assessment method. Pre- and post- assessment scores of self-reported confidence levels.	No significant difference between pre- and post- test self-reported confidence levels. Assessment data was able to inform curricular mapping. 89% of students found the assessment useful.	Single site. Details about assessment methodology poorly described.	N	N	N
Hanna, L-A. et al. (2017) [61]. Belfast, UK 11/18 (MERSQI)	Fourth-year Master of Pharmacy (MPharm) degree (n = 118). Summative and formative assessment approaches.	Aim: Establish pharmacy students’ views on assessment and an integrated five-year degree. Paper-based self-administered questionnaire. Data analysis using descriptive statistics and non-parametric tests. Open-response questions analysed using thematic analysis.	Most respondents considered formative assessment improved academic performance.	Research students were excluded from the survey.	N	N	N
Benedict, N. et al. (2017) [62] Pennsylvania, USA 15/18 (MERSQI)	First (P1, n = 111)-and third (P3, n = 108)-year undergraduate PharmD students and first-year postgraduate (PGY1, n = 25) pharmacy residents Five-station, blended simulation of the experiences of one patient, structured to correspond to Miller’s Pyramid (including knowledge and performance evaluations) administered as a progress test.	Aim: To design an assessment of practice readiness using blended-simulation progress testing. The assessment was administered to learners at various points in their professional development to gauge progress. Assessment data was analysed for differences in learner scores (P1, P3 and PGY1) for each station. Rubric validity and reliability were determined. Student perception of assessment captured using survey for P1 and P3 students.	Patterns of results were consistent with expectations that scores would improve with advancing training levels. Key performance indicators improved significantly from P1 to P3 levels and then from P3 to PGY1 levels. 40% of surveyed participants indicated that the assessment was appropriate for their level of learning, with the majority of P3 students agreeing it was appropriate.	Survey not administered to PGY1 students; low survey response rate (50%) for P3 students.	N	Y	Y
